# Modeling Paratuberculosis Transmission in a Small Dairy Herd Typical of Slovenia Suggests That Different Models Should Be Used to Study Disease Spread in Herds of Different Sizes

**DOI:** 10.3390/ani12091150

**Published:** 2022-04-29

**Authors:** Tanja Knific, Andrej Kirbiš, Jörn M. Gethmann, Jasna Prezelj, Branko Krt, Matjaž Ocepek

**Affiliations:** 1 Institute of Food Safety, Feed and Environment, Veterinary Faculty, University of Ljubljana, Gerbičeva ulica 60, 1000 Ljubljana, Slovenia; andrej.kirbis@vf.uni-lj.si; 2 Friedrich-Loeffler-Institut, Federal Research Institute for Animal Health, Institute of Epidemiology, Südufer 10, 17493 Greifswald-Insel Riems, Germany; joern.gethmann@fli.de; 3 Department of Mathematics, Faculty of Mathematics and Physics, University of Ljubljana, Jadranska ulica 19, 1000 Ljubljana, Slovenia; jasna.prezelj@fmf.uni-lj.si; 4 Department of Mathematics, Faculty of Mathematics, Natural Sciences and Information Technologies, University of Primorska, Glagoljaška 8, 6000 Koper, Slovenia; 5 Institute of Mathematics, Physics and Mechanics, Jadranska ulica 19, 1000 Ljubljana, Slovenia; 6 Institute of Microbiology and Parasitology, Veterinary Faculty, University of Ljubljana, Gerbičeva ulica 60, 1000 Ljubljana, Slovenia; brane.krt@vf.uni-lj.si (B.K.); matjaz.ocepek@vf.uni-lj.si (M.O.)

**Keywords:** *Mycobacterium avium* subsp. *paratuberculosis* (MAP), compartmental model, small dairy herds, within-herd transmission

## Abstract

**Simple Summary:**

Paratuberculosis is widespread in cattle throughout the world and is an economically important disease, especially in dairy cattle. It is caused by *Mycobacterium avium* subsp. *paratuberculosis* and is problematic due to complex transmission and unreliable diagnostic tests. The disease is present in Slovenia, where milk production is one of the most important sectors of agriculture. Our aim was to understand the disease spread in a typical Slovenian dairy herd of about 17 cows. To our knowledge, this is the first study specifically looking at the dynamics of disease spread in a small dairy herd. We relied on real data from the competent authorities on herd structure and data from the literature on disease spread, as well as expert opinion. The results showed that herd size has an influence on disease spread and that the prevalence in a small herd is probably lower than in larger herds because farmers tend to sell and buy animals frequently, which allows for the elimination of diseased animals from small herds. However, this can also reintroduce the disease into the herd. The results suggest that different assessments should be used to study the spread and control of this disease in herds of different sizes.

**Abstract:**

This study aimed to investigate the possible dynamics of paratuberculosis or Johne’s disease in a typical Slovenian dairy herd of about 17 cows. Paratuberculosis is a worldwide endemic disease of cattle caused by *Mycobacterium avium* subsp. *paratuberculosis* (MAP) and is associated with significant economic losses. We developed a stochastic compartmental model with two pathways of disease progression, infections of adult cows and infections of young animals through horizontal and vertical transmission, and transmission through animal movements. The average proportions of subclinically and clinically infected cows were 4% and 0.47%, respectively. The prevalence within the herd, which included latently infected animals, averaged 7.13% and ranged from 0% to 70.59%. Under the given circumstances, the results showed a relatively high rate of spontaneous elimination (0.22 per herd per year) of the disease and a high rate of reinfection (0.18 per herd per year) facilitated by active animal trade. To our knowledge, this stochastic compartmental model is the first to be developed specifically to represent a small dairy herd and could apply to other countries with a similar structure of dairy farms. The results suggest that different models should be used to study MAP spread in herds of various sizes.

## 1. Introduction

Paratuberculosis or Johne’s disease is a worldwide endemic disease of cattle caused by *Mycobacterium avium* subsp. *paratuberculosis* (MAP) [1]. It has a slow course with a long incubation period and usually progresses as a subclinical infection. Some animals develop a chronic wasting disorder, lose weight, produce less milk and offspring and have a higher risk of being culled and dying prematurely [2,3,4].

Based on disease progression, MAP detectability and testing targets, different classification systems for the health states of animals in herds with paratuberculosis have been proposed. Most of them describe three to four stages [5,6]. However, the dynamics of the disease within a herd are more complex. Smith et al. [7] developed a compartmental model with five MAP-positive health states for adult animals. They showed that infections in adult animals and two progression pathways could play an important role in disease dynamics on a dairy farm. Two different paths of progression refer to ones where animals remain asymptomatic but shed MAP in varying amounts intermittently or not at all, which is believed to be the predominant mode of progression and the one where animals become constant high shedders and show clinical signs months or years after infection [8]. It has been estimated that only 7% of infected animals reach the stage of clinical infection as the productive lifespan may be shorter than disease progression [9]. As animals age, their susceptibility decreases; nevertheless, infections in adult cattle occur more frequently and may be more important for the persistence of the disease in a herd than previously thought [10,11,12].

Animals in the usually long latent phase can shed small amounts of MAP in their feces and even more in the subclinical phase [13], while clinically infected animals can excrete up to 10^9^ CFU per gram of feces [14]. Fecal-oral transmission is the most common transmission route within the herd [2]. MAP in the environment of barns and pastures can serve as a source of infection for wild animals, spreading the disease to other herds [15,16]. However, the main factor contributing to the spread between herds is thought to be the movement of infected animals [17], particularly subclinically infected ones [18]. The highest risk for the spread of MAP among cattle is thought to come from cattle rather than other reared species [19].

The cost of this disease and its control in the cattle industry is high [20], and even subclinically infected animals are linked with financial losses [21]. The average loss per animal in an infected herd has been estimated at around €20–50 per year [22,23], while a clinically infected cow can cost over €200 per year [24]. In addition, MAP is suspected to be linked to Crohn’s disease [25] and some other chronic diseases in humans [26,27]. Paratuberculosis is thought to be widespread in Slovenia [28,29] but has historically been shown to be less prevalent than in other countries where dairy farming is an integral part of agriculture [29,30,31,32]. Researchers suggest that this is partly due to the structure of herd size, as small family farms characterize Slovenian cattle farming, and disease spreads more easily within than between herds [30]. The last study on the prevalence of MAP in Slovenia in 2008 estimated the true prevalence at the herd and animal levels to be about 19% and 4%, respectively [29].

Estimates of prevalence within a herd vary considerably between and within countries [31]. Still, even if the mortality in a herd remains low for years, half of the animals may be subclinically infected [33]. The highest prevalence is reported in dairy cattle, where estimates of infected herds range from 20% to 80% per country for many major milk-producing countries [33]. A recent study by Whittington et al. [32] reported that of 48 countries that participated in the survey, about half had more than 20% of herds infected. In some developed countries (e.g., Italy, France, Germany, United Kingdom), prevalence exceeded 40%. Although the reported levels of prevalence were high, the authors cautioned that both under-reporting and underestimation of paratuberculosis prevalence are common. Furthermore, comparing the prevalence between countries is difficult due to different definitions of prevalence, different diagnostic tests (which may be inadequate and sometimes have unknown sensitivity) and the selection of different samples and animals examined [31,32].

However, based on data from other countries and a large volume of import of live animals from abroad [34], we hypothesized that with the increase in average herd size in Slovenia in recent years, MAP prevalence within the herd has also increased. The aim of this study was to investigate the possible disease dynamics within a typical Slovenian dairy herd, to estimate the likely prevalence within the herd and the proportion of subclinically and clinically infected cows. For this purpose, we developed a compartmental model of MAP spread within the herd in Slovenia. We chose this model because it allowed the use of demographic data, data on the movement and production of Slovenian cattle provided by different agencies and published data from empirical studies or modeling studies on the epidemiological characteristics of MAP. Moreover, in both cases, the data were supported by expert opinions. Furthermore, the model enabled the identification of the parameters with the highest impact on disease spread.

## 2. Materials and Methods

### 2.1. Structure of the Model

We developed a stochastic compartmental model of MAP spread in a typical Slovenian dairy herd. The typical herd was defined as a herd with an average number of dairy cows on a dairy herd in Slovenia, comprising 17.1 cows, 8.4 heifers and 5.6 female calves in 2018 [35]. Most of these small farms are family-owned [36] and raise other species besides cattle [37]. The cattle are mostly kept in stables, but in the Mediterranean, pre-Alpine and Alpine regions, they have access to pastures [38]. In Slovenia, the grazing season starts in April and lasts until mid-November [34], although the duration depends on the region [38]. Dairy cattle in 2018 were mainly Holstein Friesian (36.1%) and Simmental (29.1%) breeds, followed by Brown Swiss (9.8%), Simmental crossbreeds (8.5%) and the autochthonous Cika breed (0.1%), while others were multiple crossbreeds or of unknown origin (16.4%) [36].

The model was developed using the Epidemiological Multi-Level Simulation Framework (EMULSION), version 1.0.12 [39], which employs finite state machines. In the present study, the state machine was defined by the evolution of health states or compartments, as they are called in epidemiological models. The transitions of individual animals between the compartments were described with different rates, which were automatically converted into probabilities, thus introducing stochasticity.

**Figure 1:**
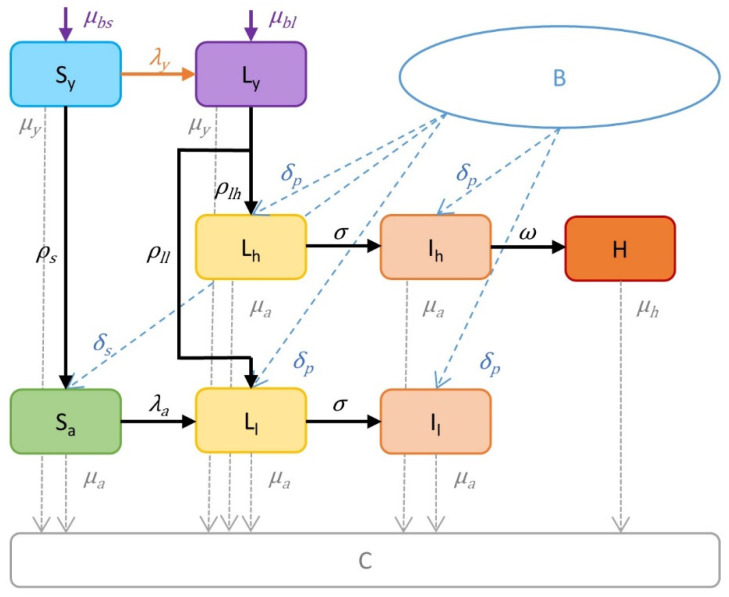
Flow diagram of the model for the spread of *Mycobacterium avium* subsp. *paratuberculosis* in a typical Slovenian dairy herd. Possible health states represented by boxes are susceptible young (*S_y_*); latent young (*L_y_*); latent cows (*L_h_*), infected cows (*I_h_*) and high shedding cows (H) on the high shedding pathway; susceptible cows (*S_a_*); latent cows (*L_l_*) and infected cows (*I_l_*) on the low shedding pathway; and culled, dead or sold animals (C). The bought compartment (B) represents animals outside the herd that can be introduced. The arrows represent how individual animals change from one state to another. All parameters and calculations are defined in Table 1 and Table 2.

To capture the epidemiological characteristics of MAP, we used several compartments for adult cows and young animals. The model’s basic structure is shown as a flow chart in Figure 1. Adult cows were divided into one susceptible state and five MAP-positive states as we modeled two different infection pathways, namely, the low shedding pathway with the compartments of susceptible cows (*S_a_*), latent cows (*L_l_*) and infected cows (*I_l_*), and the high shedding pathway with the compartments latent cows (*L_h_*), infected cows (*I_h_*) and high shedders (H). Only animals infected as calves could enter the high shedding pathway, but they could also progress through the low shedding pathway. Animals infected as adults, on the other hand, could only enter the low shedding pathway. We added two compartments for young animals: susceptible young (*S_y_*) and latent young (*L_y_*), to model the effects on reproduction and infections on calves and heifers. We considered infected cows on both pathways as subclinically infected and we considered latent young and cows as animals showing no signs of the disease. High shedding cows were considered clinically infected.

To record the culling or mortality of animals and the admission of new animals into the herd, we added two further compartments. Culled, dead or sold animals were moved to the culled compartment (C) and were no longer counted, whereas the bought compartment (B) counted animals and initially contained 1000 animals. The latter was an arbitrary figure and stood for animals outside the herd needed to calculate the number of animals that could be purchased and brought into the herd.

### 2.2. Model Parameters, Calibration and Simulation

First, we created a basic model, wrote down all the changes between the compartments, defined the rates and, where necessary, the calculations of the rates. Then we implemented the parameter values for the average dairy herd in Slovenia based on data obtained from the Agricultural Institute of Slovenia [35], the Results of Dairy and Beef Recording [36] and the Statistical Office of the Republic of Slovenia [40]. All data used referred to the year 2018. The initial herd consisted of 17 susceptible dairy cows (*N_i_*) and 15 susceptible young animals (*Y_i_*). The parameters estimated based on the Slovenian data were the birth rate (*µ_b_*), culling rates (*μ_a_*, *μ_h_*), herd renewal by own young animals (*ρ_s_*) or by the introduction of new animals (*δ*) and the animal-level paratuberculosis prevalence (*P*) for animals that can be introduced into the herd. We calibrated the model to stabilize the herd size for at least 20 years.

The parameters for MAP transmission, incubation, disease progression and birth rates of infected animals were taken from the literature [9,41,42,43,44,45]. These parameters were estimated either from the empirical data [9,41,43,44,45], by calibrating models similar to ours or from other models developed by different authors [41,42,43], so we recalculated some values. First, we considered the values on which several authors seemed to agree. If this was not possible, we chose the value corresponding to the worst-case scenario. Then, we repeated the calibration of the model to keep the herd size stable for at least 10 years. Based on expert opinion, we adjusted the values of the culling rates. All parameter values of the model were deterministic. The parameter definitions, their values and sources are listed in Table 1. The calculations of the parameters and transitions are shown in Table 2.

**Table 1:** Parameters of the model for the spread of *Mycobacterium avium* subsp. *paratuberculosis* in a typical Slovenian dairy herd.

Symbol	Description	Value	Source
*N_i_*	Initial number of adult animals; dairy cows	17	[35]
*Y_i_*	Initial number of young animals; calves and heifers	15	[35]
σ	Transition rate from latent to infected cows (/day)	0.00274	[9,41]
ω	Progression rate from infected cows on high shedding pathway to high shedding cows (/day)	0.00137	[9,41]
$\beta_{l}$	Transmission rate for latently infected cows (/day)	0.000027	[42,43]
$\beta_{i}$	Transmission rate for infected cows (/day)	0.000274	[43]
$\beta_{h}$	Transmission rate for high shedding cows (/day)	0.00274	[43]
$\mu_{a}$	Culling rate for all cows except high shedding cows (/day)	0.000457	Expert opinion
$\mu_{h}$	Culling rate for high shedding cows (/day)	0.003653	Expert opinion
$\mu_{r}$	Culling rate or sale of surplus young animals (/day)	0.000322	Expert opinion
δ	Rate of buying in new animals (/day)	0.000009	[34]
P	Prevalence at animal level	0.08	[4,29]; expert opinion
$\mu_{b}$	Birth rate for all cows except high shedding cows (/day)	0.001315	[36]
$\mu_{bh}$	Birth rate for high shedding cows (/day)	0.000411	[44]
$\gamma_{p}$	Proportion of latent calves per latently infected and infected cows	0.25	[45]
$\gamma_{h}$	Proportion of latent calves per high shedding cows	0.65	[41]
$\beta_{yy}$	Transmission rate from latently infected calves to susceptible calves (/day)	0.000007	[43]
$\beta_{yl}$	Transmission rate from latently infected cows to susceptible calves (/day)	0.00021	[43]
$\beta_{yi}$	Transmission rate from infected cows to susceptible calves (/day)	0.002099	[43]
$\beta_{yh}$	Transmission rate from high shedding cows to susceptible calves (/day)	0.020986	[43]
$\beta_{hh}$	Transmission rate from latently infected heifers to susceptible heifers (/day)	0.000003	[43]
α	Proportion of latently infected young animals entering high shedding pathway	0.335	[44]

**Table 2:** Calculation of parameters or transitions in the model for the spread of *Mycobacterium avium* subsp. *paratuberculosis* in a typical Slovenian dairy herd.

Symbol	Description	Calculation
N	Number of adult animals; dairy cows	$N=S_{a}+L_{l}+L_{h}+I_{l}+I_{h}+H$
Y	Number of young animals; calves and heifers	$Y=S_{y}+L_{y}$
$\lambda_{a}$	Force of infection for adult animals (/day)	$\lambda_{a}=\frac{\beta_{l}(L_{l}+L_{h})+\beta_{i}(I_{l}+I_{h})+\beta_{h}H}{N}$
$\delta_{s}$	Transition rate from bought to susceptible cow compartment	$\delta_{s}=\delta \left(1-P\right)$
$\delta_{p}$	Transition rate from bought to latently infected and infected cow compartments	$\delta_{p}=\frac{\delta \cdot P}{4}$
$\mu_{bl}$	Birth rate of latently infected female calves and direct transmission from mothers	$\mu_{bl}=\mu_{b}\gamma_{p}\left(L_{l}+L_{h}+I_{l}+I_{h}\right)+\mu_{bh}\gamma_{h}H$
$\mu_{bs}$	Birth rate of susceptible female calves	$\mu_{bs}=\mu_{b}S_{a}+\mu_{b}\left(1-\gamma_{p}\right)\left(L_{l}+L_{h}+I_{l}+I_{h}\right)+\mu_{bh}(1-\gamma_{h})H$
$\lambda_{y}$	Force of infection for young animals (/day)	$\lambda_{y}=(\beta_{yy}+\beta_{hh})\frac{L_{y}}{Y}+\frac{\beta_{yl}(L_{l}+L_{h})+\beta_{yi}\left(I_{l}+I_{h}\right)+\beta_{yh}H}{N+\frac{Y}{2}}$
$\rho_{s}$	Replacement of culled animals with own animals; from *S_y_* to *S_a_*	$\rho_{s}=(N_{i}-N)\frac{2}{Y}$
$\rho_{ll}$	Replacement of culled animals with own animals; from *L_y_* to *L_l_*	$\rho_{ll}=\rho_{s}\left(1-\alpha \right)$
$\rho_{lh}$	Replacement of culled animals with own animals; from *L_y_* to *L_h_*	$\rho_{lh}=\rho_{s}\cdot \alpha$
$\mu_{y}$	Culling, mortality or sale of young animals (/day)	$\mu_{y}=\{\begin{array}{c} \mu_{r},\;\;if\;Y-Y_{i}>0\\ 0,\;\;otherwise \end{array}$

We simulated MAP spread within the herd over 10 years with 1000 iterations and a daily time step. The simulation began with the introduction of one infected animal into the naive herd, namely an infected cow on the high shedding pathway (*I_h_*). Based on the prevalence at the animal level (*P*), the additional infected cows could be introduced into the herd through the bought compartment (B). This meant that more than one infected cow could be introduced into the herd or that multiple reinfections could occur in a herd where the disease was spontaneously eliminated. This was taken into account with the rate of buying in new animals (*δ*) and calculated so that each year, about 3.1 cows were introduced into the herd from the bought compartment (B). The number of bought animals was based on the average value of the local network measure, the so-called weighted in-degree, obtained in the network analysis study of cattle movements in Slovenia conducted by Knific et al. [34]. The weighted in-degree of a farm is essentially the number of cows moved from other farms to the specified farm. The health states of a bought animal depended on the prevalence at the animal level (*P*). We assumed that the purchased animals were adults and could be of any health state, except for high shedders, i.e., we assumed that farmers would not buy clinically affected cows. Therefore, to define the transition rate from the bought compartment to other MAP-positive compartments (*δ_p_*), we divided the transition rate from the bought compartment into four MAP-positive compartments. We estimated the prevalence at an animal level using the results of two studies conducted in Slovenia and expert opinion.

The rate of susceptible cows becoming latent cows on the low shedding pathway was defined by the force of infection (*λ_a_*), which depended on the number of cows in each health state and the different degrees of transmission potential for cows in each state. The transmission rates for cows in different health states (*β_e_, β_i_, β_h_*) were taken from the results of published models and recalculated to fit the time step of this model. It was assumed that the transition from a latently infected cow to an infected cow on both pathways took up to one year. In the model, this was defined by the incubation rate (*σ*). Within two years, infected cows on the high shedding pathway could become high shedders showing clinical signs, defined by the rate of progression (*ω*). The culling rate of adults (*μ_a_*) was derived from the assumption that cows reach six productive years in a typical Slovenian dairy herd. We assumed that farmers cull clinically affected animals within 0.75 years, defined as the culling rate of cows with high shedding (*μ_h_*). Meanwhile, the culling rate of young animals (*μ_y_*) was calculated to maintain the herd size throughout the simulation period. In the model, we called this the culling of young animals, but in the real world, this could be the culling or mortality of young animals or even the sale of surplus young animals.

Renewal of the herd was taken into account by adding the bought compartment and by replacement from the own young stock. Young animals were born at a rate that depended on the birth rate of female calves per cow (*μ_b_*). We assumed that only the cows with high shedding had a lower birth rate (*μ_bh_*). Calves can be born susceptible (*S_y_*) or already latently infected (*L_y_*). The probability of calves being born infected or calves being infected directly from their mothers after birth was different for latently infected cows and infected cows (*γ_p_*) on the low and high shedding pathways and for the high shedders (*γ_h_*). In addition, susceptible young animals can become infected from other animals in the herd, which was taken into account in the force of infection for young animals (*λ_y_*). Since we modeled calves and heifers together as young animals, we calculated their force of infection, assuming that each of them represented half of the young animals in the herd. As can be seen from the equation in Table 2, we assumed that half of the young animals, namely the calves, could become infected from all other animals in the herd, while the other half of the young animals, namely the heifers, could become infected only from the heifers. The progression of young animals into adults was calculated in such a way that the size of the herd remained stable. Susceptible young animals became susceptible cows, while latently infected young animals became latently infected cows on the pathways of low or high shedding. The proportion of latently infected young animals taking the high shedding pathway was determined by *α*.

### 2.3. Model Outputs and Validation

The final step in model development was internal validation, which consisted of a thorough review of all underlying assumptions, calculations and parameters. The initial model assumptions and parameters proposed by T.K. were based on available census data for Slovenian herd characteristics and literature for MAP epidemiological characteristics. However, not all the studies reviewed are presented as a formal review of the modeling studies is beyond the scope of this study. The studies that were used to determine the parameters for the present model are discussed in more detail in the discussion. The initial assumptions and parameters were reviewed by M.O., a leading expert on paratuberculosis in Slovenia. Throughout the development and analysis of the model, the underlying assumptions, calculations and parameters were reviewed several times by J.M.G., a recognized specialist in epidemiology. In addition, the model calculations and assumptions were reviewed by a mathematician, J.P. Finally, the developed model, assumptions and results were reviewed by a second Slovenian senior researcher with experience in paratuberculosis diagnostics, B.K. Furthermore, the model itself, the results and their interpretation were discussed with all co-authors and compared with other studies modeling MAP transmission within the herd.

The outcomes we were interested in were the number of dairy cows, prevalence, number of subclinically and clinically infected cows, spontaneous elimination of the disease and occurrence of reinfection. Prevalence considered all infected dairy cows in five different compartments, while subclinically infected cows were those in infected compartments on both pathways. Clinically infected cows were those represented in the high shedder compartment. We reported the number of susceptible and latently infected young animals. Still, due to the adjustments in modeling the number of young animals described above, we did not include the latently infected young animals in the prevalence calculations. Spontaneous elimination of disease was defined as the absence of animals in any of the five MAP-positive health states for cows due to culling, mortality or sale of animals, and after that, reinfection was detected if at least one animal was recorded in any of these five compartments.

The second part of the validation of the model was a sensitivity analysis. It was performed for the epidemiologically most important parameters with the minimum and maximum values estimated by an expert to assess the robustness of the model. We tested the sensitivity of these parameters by running the model 16 times with 1000 iterations each for the minimum and maximum values of the chosen parameters, fixing all parameters except the one tested to see their effect. The values used in the sensitivity analysis are shown in Table 3. The maximum value for the transition rate from a latent to an infected cow (σ) and the progression from an infected to a high shedding cow (ω) are smaller than the values used in the model because they are calculated based on expert opinion on how long the individual animal can remain in the latent or infected compartment. To be precise, the expert estimated that a cow could remain latently infected for a maximum of three to four years and infected for up to five years. The minimum value of zero for these two parameters means that a cow never progresses into an infected or high shedding state.

We analyzed the results from EMULSION using the R programming language, version 4.1.2 [46]. The results are presented with the basic descriptive statistics of the outcomes of interest: minimum, maximum, mean, standard deviation (SD), median and percentiles (PC). Due to the non-normal distribution of the variables, correlations were calculated using Spearman’s rank correlation coefficient, with *p*-values adjusted by Holm’s method to account for multiple comparisons. The threshold for a significant result was a *p*-value < 0.05.

## 3. Results

### 3.1. Distribution of Animals in Different Health States

**Figure 2:**
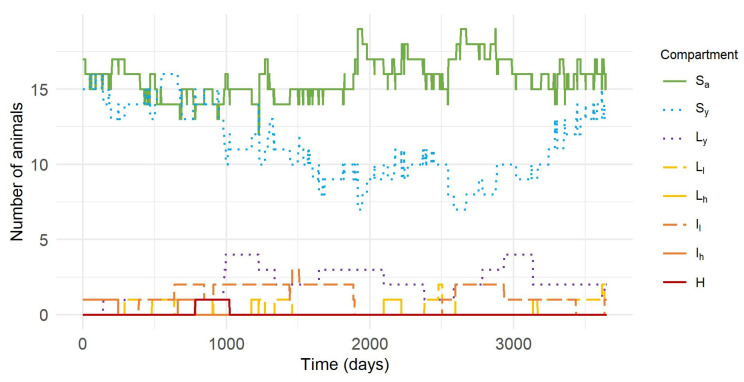
Demonstration of an iteration of the model simulations for the spread of *Mycobacterium avium* subsp. *paratuberculosis* in a typical Slovenian dairy herd. Susceptible cows (*S_a_*); susceptible young (*S_y_*); latent young (*L_y_*); latent cows on low shedding pathway (*L_l_*); latent cows on high shedding pathway (*L_h_*); infected cows on low shedding pathway (*I_l_*); infected cows on high shedding pathway (*I_h_*); high shedders (H).

To illustrate how compartmental model simulations are carried out, an iteration of the stochastic simulation is shown in Figure 2. As can be seen from this simulation, the number of cows per herd changed over time. One thousand iterations of the model throughout the study period resulted in an average of 17.49 cows per herd, with a minimum of 10 and a maximum of 28 cows per herd. However, both extreme values were outliers; the 5th PC was 17 and the 95th PC was 19 cows per herd. The variations in herd size were the reason why the results were primarily expressed in proportions and not absolute values.

**Figure 3:**
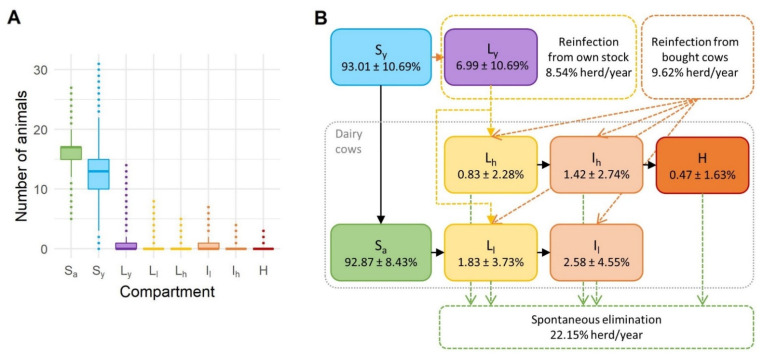
Distribution of animals in individual health states in the model for the spread of *Mycobacterium avium* subsp. *paratuberculosis* in a typical Slovenian dairy herd. (**A**) Number of animals in individual health states. (**B**) Flow diagram of the model showing the proportion of cows in each health state out of the total number of dairy cows in a herd and the proportion of young female calves and heifers in the two young animal states (mean ± standard deviation). The possible reinfections and spontaneous eliminations (mean per herd/year) are marked with dashed lines. Susceptible cows (*S_a_*); susceptible young (S_y_); latent young (*L_y_*); latent cows on low shedding pathway (*L_l_*); latent cows on high shedding pathway (*L_h_*); infected cows on low shedding pathway (*I_l_*); infected cows on high shedding pathway (*I_h_*); high shedders (H).

**Figure 4:**
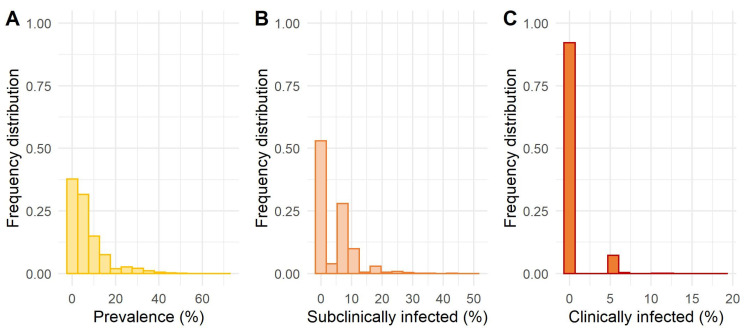
Frequency distribution of (**A**) the prevalence of *Mycobacterium avium* subsp. *paratuberculosis* within the herd and (**B**) the proportions of subclinically infected cows and (**C**) clinically infected cows.

Figure 3 shows the proportion of animals in each health state per herd over the entire simulation period and for all simulations. The number of susceptible young animals with a mean value of 12.45 per herd and 3.67 SD varied the most. Again, the extreme values were outliers; the 5th PC was six and the 95th PC 18 susceptible young animals per herd. The mean value of latent young animals was 0.96 per herd (1.49 SD). For all MAP-positive health states in cows, the median value was zero, while the median value for susceptible cows was 94.44% or about 16 cows. In none of the simulations was the entire herd infected. The minimum percentage of susceptible cows was 29.41%, and the maximum was 100%, which means that paratuberculosis was eliminated in some cases. The mean values for other health states were less than one percent for a latently infected cow on the high shedding pathway and high shedders or clinically infected cows. On average, there were fewer than two percent of infected cows on the high shedding pathway and latently infected cows on the low shedding pathway, and there were fewer than three percent of infected cows on the low shedding pathway. For infected cows on both pathways and subclinically infected cows, the mean was 4% (5.42% SD) with a median of zero. The maximum subclinically and clinically infected cows were 50% and 18.75% per herd, respectively. The 95th PC was one cow per herd in the high shedding pathway for MAP-positive health states and two cows in the low shedding pathway. Altogether, all these compartments gave a mean prevalence within the herd of 7.13% (8.43% SD), with the 95th PC being 23.53% and the maximum prevalence 70.59%. The frequency distributions of prevalence, subclinically infected and clinically infected cows are shown in Figure 4. Clinically infected animals were observed in 67.5% of the simulated herds and were present in the herd for an average of 382.36 days (378.55 days SD) and a maximum of 2707 days.

**Figure 5:**
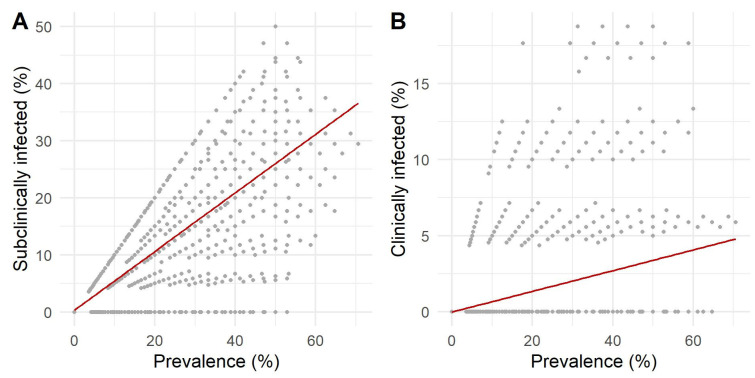
Correlations between the prevalence of *Mycobacterium avium* subsp. *paratuberculosis* within the herd and (**A**) the proportion of subclinically infected cows and (**B**) clinically infected cows per herd.

**Figure 6:**
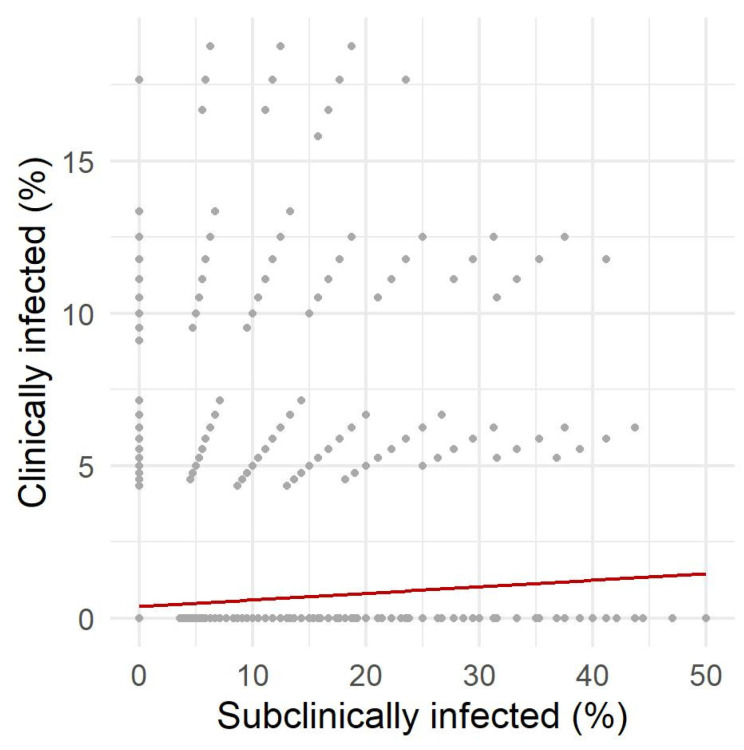
Correlation between the proportion of subclinically and clinically infected cows in a *Mycobacterium avium* subsp. *Paratuberculosis*-positive herd.

The correlations between the proportions of clinically and subclinically infected cows and prevalence were all highly statistically significant (adjusted *p*-values < 0.0001; Figure 5 and Figure 6). Spearman’s rank correlation coefficient showed a strong correlation between subclinical cows and prevalence (Spearman’s ρ = 0.79). In contrast, the correlation between the proportion of clinical cows and prevalence was weak (Spearman’s ρ = 0.31). Interestingly, the correlation between the proportion of clinical and subclinical cows was very weak (Spearman’s ρ = 0.04).

### 3.2. Occurrence of Spontaneous Elimination and Reinfection

Out of 1000 simulated herds, only 114 remained infected over the entire 10 years of the study period. On average, the herds were infected for 2275 days (1093 SD). The minimum length was 11 days. Under the given circumstances, the incidence of spontaneous elimination of the disease from the herd was 0.22 per herd per year, while the incidence of reinfection was 0.18 per herd per year. During the 10 years, several spontaneous eliminations and reinfections occurred in most herds. More than one spontaneous MAP elimination occurred in 676 herds, and 553 herds were reinfected more than once. The maximum values were eight eliminations in one herd and seven reinfections in two different herds. The source of reinfections in dairy cows were replacements from their own young animals in 47.03% of the cases, and the rest were caused by animals newly introduced into the herd. 2610 animals introduced into the herds were infected, corresponding to a prevalence of 8.12% at the animal level. In total, 1648 of the bought infected animals were introduced into already infected herds.

### 3.3. Sensitivity Analysis

In the sensitivity analysis, we investigated the effect of changing eight epidemiologically significant parameters on the herd prevalence of MAP and the proportions of subclinically and clinically infected cows per herd (Table 4, Table 5 and Table 6). Overall, the results were in line with the expectations. The parameters with their minimum and maximum values that had the most significant influence on the simulation results were the transmission rate from infected cows to young animals (*β_yi_*) and the transmission rate of infected cows (*β_i_*). The minimum values of these two parameters resulted in fewer infected cows, while the maximum values more than doubled the prevalence within the herd. Other parameters with noticeable effects were the prevalence at the animal level (*P*), transmission rate from high shedders to young animals (*β_yh_*) and transmission rate for high shedders (*β_h_*). In addition, the progression from an infected cow on the high shedding pathway to a high shedder (*ω*) had a significant influence on the proportion of clinically infected cows. The transition rate from a latently infected to an infected cow (*σ*) and the proportion of latently infected young animals entering the high shedding pathway (*α*) had little influence on the results.

## 4. Discussion

We developed a stochastic compartmental model to estimate the possible proportion of subclinically and clinically infected cows in a typical Slovenian dairy herd infected with MAP. The main reason for developing the model was the lack of data on MAP prevalence within the herd in Slovenia, as the last study was conducted in 2008 in three dairy herds [4]. Another problem, besides the small sample size and the timing of the study, was that the prevalence was studied in large herds, which do not represent the structure well of dairy herds in Slovenia. In a study on prevalence at the herd and animal levels, Ocepek et al. [30] argued that the low prevalence at the animal level in Slovenia could be partly explained by the existence of numerous small herds, as spread within herds is more likely than between herds. We hypothesized that since the average size of farms in Slovenia has increased in recent years, mainly because farmers with small herds have given up farming, this has created better conditions for MAP to spread.

We defined a typical dairy herd in Slovenia as a herd with the average number of dairy cows in a dairy herd in Slovenia. All possible demographic parameters of the herd were estimated from Slovenian data. Some of the values were corroborated with expert opinion because the average value does not reflect the actual conditions in a small herd. This is because the average values are calculated at the animal level, which biases them toward larger herds. For example, farm data by size class of the number of dairy cows for 2016 showed that dairy herds with 20 or more cows per herd accounted for less than 17% of all dairy herds but raised almost 54% of all dairy cows in Slovenia [40]. Thus, the average values at the animal level better reflect the situation in larger herds than in smaller herds. The average number of dairy cows per farm was 17.1, so we used 17 cows as the initial number of dairy cows in the compartmental model. However, we did not use the average number of young animals per herd in Slovenia, but the average number of young animals in herds with 17 cows. According to the Results of Dairy and Beef Recording [36], dairy cows’ average performance lifespan when culled in 2018 was about 1318 days. The expert’s opinion was that this is more reflective of highly productive herds, while smaller herds are less intensive and farmers tend to keep their cows longer. The estimated lifespan of a cow in a small herd was eight years, i.e., six productive years, which was the value on which we based the culling rate for all cows except high shedders. The culling rate for the high shedders was calculated based on the expert opinion that farmers with smaller herds tend to treat their cows longer and hope for improved health than those who run more intensive herds. The number of cows added to the herd and the birth rate of all cows, except for high shedders, were calculated directly from the available data. We tried to make some adjustments to these figures to reflect the situation in a small herd better. In none of these cases did the herd size remain stable over the entire period studied when MAP infection was introduced, as the introduction of MAP infection increased the culling rate and decreased the birth rate. Therefore, we decided to calibrate the model to ensure that the herd size of a naive herd is stable for at least 20 years. Finally, we used the values calculated directly from the available data after calibrating the model with MAP infection. In addition, the culling rate of young animals of about 12% per year was only used if there were surplus young animals in the susceptible young and latent young compartments, to ensure that there were enough replacement cows to keep the number of adult cows stable. Additionally, the developed model was simulated with an initial herd size set at 100 dairy cows to test whether the same model could be used to study the spread in larger herds. It turned out that this was not the case as the model was not stable. However, the aim was not to find a threshold for herd size in this model but to determine the possible proportions of subclinically and clinically infected cows in a typical Slovenian herd.

Other parameters used relating to transmission of MAP, disease progression and its impact on reproduction and culling were based on data from the literature. We chose the compartmental model because, on the one hand, a whole series of studies on the dynamics of MAP infections within the herd have been published, but on the other hand, many parameters have only been determined by model calibrations and expert opinions. We assumed that modeling at the compartmental level with introduced stochasticity would be sufficient to gain insight into the MAP within-herd dynamics and allow for the use of published parameters from different types of models. Indeed, we collected parameters from compartmental models and individual or agent-based models with varying combinations of health states and transitions, infection pathways, herd dynamics considered and time steps. Daily, weekly or monthly time steps were used in these studies. We used the parameters from the following studies, although we had to recalculate some values to fit the structure of our model and its time step.

Marcé et al. [41] developed a stochastic compartmental model of the spread of MAP within a dairy herd with six health states, namely susceptible, transiently infectious, latently infected, subclinically infected, clinically affected and resistant. They modeled the herd dynamics over a 25-year period with representative barn facilities. The resistant animals were all uninfected animals older than one year, and they did not consider infections in adult cows. They used data from French agricultural statistics to construct a herd. Their original herd included 114 dairy cows. However, in another study using the same model, they also simulated MAP spread in a herd with 35 adult animals but found no correlation between the herd size and outcomes [47]. Nevertheless, this does not represent a typical herd in Slovenia well, which in 2018, was only half the size of the smallest herd they used in the simulation study. Plus, they only considered vertical and indirect transmission from calf to calf and from cow to calf. In their study, the prevalence in adult cows reached 87%, 67% and 15% of infected, infectious and affected animals, respectively.

All other studies from which the parameter values for this study were taken were designed for MAP spread within dairy herds in the United States [9,42,43,44,45]. Their herd sizes varied from 100 to 1000 dairy cattle. They all considered direct, indirect and vertical transmission, and adult infections, but implementations varied. Except for Martcheva et al. [45], who modeled only the calf and cow compartment, all other researchers modeled heifers as a separate group or individuals. Their study had a very different focus than the model presented here, as they were interested in the mechanisms of the disease, and therefore, developed a MAP within-host model linked with an epidemiological model.

Robins et al. [42] and Al-Mamun et al. [43] developed individual, or agent-based, models with three infection states: latent or exposed, low shedding cow and high shedding cow. Both used some parameters from the literature but took different approaches to obtain the missing parameter values for MAP transmission. Robins et al. [42] used expert opinion and assumptions, while Al-Mamun et al. [43] assumed the prevalence and obtained transmission coefficients and rates by calibrating the model. The aim of the study by Robins et al. [42] was to evaluate the cost-effectiveness of control measures. The model showed that the initial paratuberculosis prevalence on the farm increased from 33.1 ± 0.2% to 87.7 ± 1.7% during the 10-year simulation. Al-Mamun et al. [43] investigated the performance of four test-based control strategies. Three MAP prevalence scenarios were simulated: 5–15%, 15–25% and 30–40%. After 15 years, this resulted in simulated true prevalence levels of 10.2%, 22.5% and 34.7%, respectively. The model results suggested that risk-based culling can reduce the prevalence but cannot eliminate the disease. The results of our model indicate a similar outcome if culling of clinically infected cows is implemented.

Mitchell et al. [9] developed a state transition model with transient, latent, low shedder and high shedder states for infected states. They used longitudinal field data with the considered MAP strains to estimate the parameter values. The model showed a MAP prevalence of about 25% in adult animals, although field data indicated a prevalence of about 20%. They suggested that even using an extensive field dataset is likely to underestimate the true prevalence and incidence due to inaccurate testing and slow disease progression. Smith et al. [44] modeled two pathways of disease progression, which formed the basis for developing our model. They examined the economic implications of paratuberculosis control over a 25-year period in herds of 100 and 1000 dairy cattle with endemic paratuberculosis at an initial prevalence of 10% or 20%. The results showed that with the control measures implemented, elimination only occurred in a smaller herd with lower initial prevalence, and no spontaneous elimination was observed. The main interest of this study was the number of subclinically and clinically infected cows. The authors modeled calves and heifers together as young animals, and since their transmission potentials are different, we calculated the force of infection for young animals accordingly.

In a study examining the control of paratuberculosis in 48 different countries, the most commonly reported prevalence in dairy herds was 5–15% [32]. However, as mentioned above, many studies point to the problem of inadequate diagnostic tests and the iceberg phenomenon of paratuberculosis infections. Some estimate that up to 70% of infected animals are undetectable [5]. Contrary to what we expected based on prevalence and modeling studies, our model did not show a higher prevalence of MAP within the herd in Slovenia than previously estimated [29]. Even though the average herd size has increased from 12.5 dairy cows in 2008 to 17.1 dairy cows per farm in 2018, this has not created better conditions for MAP spread. Our results have shown that the movements of animals out of and into herds and the prevalence in a region are most important for the MAP status of a small herd. In a previous study, we found that most Slovenian cattle farms are very active in the animal trade, so we included the introduction of new animals into the herd based on real data [34]. In the present study, we confirmed the findings of Beaunée et al. [48], who found that in an enzootic situation, similar to the one presented in this study, the risk of infection quickly becomes high for farms that purchase more than three animals per year. We modeled the introduction of about 3.1 animals per year, resulting in a 26.1% probability of introducing an infected animal into the herd in one year, giving a reinfection rate of 0.1 per herd per year, as some of these animals were introduced to already infected herds. Reinfection by own young animals contributed 0.09 to the annual reinfection rate per herd. On the other hand, the active trade, slow progression of the disease and small size of the herd also allowed for a high rate of spontaneous elimination of the infection from the herd (0.22 per herd per year). This led to a median within-herd prevalence of 5.56% in the simulated farms and may explain why the prevalence in Slovenia is lower compared to countries with larger farm sizes. The high rate of spontaneous elimination of MAP was also observed in a study by Marcé et al. [41], but they started the infection with one infected animal being introduced into the herd and did not consider reinfections of the herd by introducing additional animals. This resulted in 29.5% of herds being persistently infected, whereas we considered reinfections possible and only 11.4% of the simulated herds remained infected throughout the entire study period. In this study, we started the infection with one infected animal, and one herd was free of the disease after only 11 days.

The major pitfall of this modeling study was the random selection of cows to be introduced into the herd, as farmers in Slovenia purchase animals from the local community or other countries and regions that may have a different epidemiological situation. Not all herds from which animals are purchased have the same risk of having infected animals. The second important pitfall in the model was the parameters used to describe the MAP epidemiological characteristics and consequences of infection. However, the logical results of the sensitivity analysis attested that we appropriately calibrated the input parameters and that the model was mathematically sound. Nevertheless, the parameters based on expert opinion used to describe the culling rates and prevalence of MAP at the animal level introduced a bias into the model that can only be eliminated by collecting empirical data. In addition, the model contained a number of underlying assumptions that likely contributed to a bias of the within-herd transmission potential. As with any compartmental model, there was no differentiation between animals in a specified compartment based on individual characteristics such as age, breed or other factors associated with immunocompetence. The model does not assume any specific breed of dairy cows. The compartmental model also fails to take into account the time an animal has spent in an individual compartment. Therefore, the risk of disease transmission or progression to the next state is assumed to be the same for all animals in the same compartment. Furthermore, routes of transmission other than from cattle to cattle were neglected. The main reasons for not including these parameters in the model were the lack of empirical data and the fact that transmission between cattle is considered the predominant route of transmission.

The sensitivity analysis also allowed us to examine the effects of the minimum and maximum plausible values of the eight epidemiologically most important parameters. We found that the transmission rates from infected cows to young animals and from infected cows to susceptible cows were the most significant parameters for determining the prevalence and the proportion of subclinically and clinically infected cows per herd. This means that infected cows on the low or high shedding pathway were the most influential compartments in the model. However, the transmission rate of high shedders followed closely behind, but the overall effect was minor because there were fewer clinically infected cows than infected cows. The proportion of latently infected young animals that entered the high shedding pathway had a more significant effect on the proportion of cows with high shedding than on the proportion of subclinically infected cows and prevalence. These results suggest that culling high shedding animals under the applied conditions would have only a limited effect on reducing MAP prevalence within the herd.

## 5. Conclusions

To our knowledge, this stochastic compartmental model is the first to be developed specifically to represent a small dairy herd. There have been some attempts to model small herds but these were not small for the Slovenian herd structure but rather of medium size. This work has shown that comprehensive insights into the spread of MAP can be gained despite the lack of empirical data. However, the lack of empirical data on MAP epidemiological characteristics was identified as the main gap. The results presented suggest that different models should be used to study MAP spread in herds of different sizes. The model was based on several assumptions and could be further developed; nevertheless, we believe it sufficiently represents a typical Slovenian herd. The development of this model is also important for other countries with a similar dairy system as it can be used to test the efficiency of different control measures. Furthermore, these findings also have implications for countries with generally larger dairy herds, as the different dynamics of MAP infections within a smaller herd could be taken into account in targeted control measures. This could be of great importance if a link between MAP and human diseases is established as the results could serve as decision support for MAP intervention measures.

## Figures and Tables

**Table 3:** Minimum and maximum values for the epidemiologically most important parameters used in the sensitivity analysis.

Symbol	Description	Value Used in the Basic Model	Minimum Value	Maximum Value
σ	Transition rate from latent to infected cows (/day)	0.00274	0	0.000685
ω	Progression rate from infected cows on high shedding pathway to high shedding cows (/day)	0.00137	0	0.000548
$\beta_{i}$	Transmission rate for infected cows (/day)	0.000274	0	0.00274
$\beta_{h}$	Transmission rate for high shedding cows (/day)	0.00274	0	0.027398
P	Prevalence at animal level	0.08	0.01	0.2
$\beta_{yi}$	Transmission rate from infected cows to susceptible calves (/day)	0.002099	0	0.013699
$\beta_{yh}$	Transmission rate from high shedding cows to susceptible calves (/day)	0.020986	0.00274	0.068494
α	Proportion of latently infected young animals entering high shedding pathway	0.335	0.1	0.5

**Table 4:** Results of the prevalence of *Mycobacterium avium* subsp. *paratuberculosis* within the herd from the sensitivity analysis of the epidemiologically most significant parameters. The parameters and their values in the basic model and the sensitivity analysis are defined in Table 3. Percentile, PC; quantile, Q; standard deviation, SD.

Parameter	Min./Max. Value	5th PC	1st Q	Median	Mean	SD	3rd Q	95th PC
Basic model	/	0	0	5.56	7.13	8.43	11.11	23.53
σ	Min.	0	0	5.56	6.24	7.31	10.53	22.22
	Max.	0	0	5.56	6.53	7.63	11.11	22.22
ω	Min.	0	0	5.56	5.38	6.28	5.88	17.65
	Max.	0	0	5.56	6.63	7.85	11.11	23.53
$\beta_{i}$	Min.	0	0	5.56	6.83	8.37	11.11	23.53
	Max.	0	5.26	11.76	16.88	17.32	27.78	52.94
$\beta_{h}$	Min.	0	0	5.56	6.49	7.47	11.11	22.22
	Max.	0	0	5.88	12.46	15.43	17.65	47.06
P	Min.	0	0	0	4.08	6.89	5.88	17.65
	Max.	0	5.56	11.11	12.73	11.30	17.65	35.29
$\beta_{yi}$	Min.	0	0	5.56	5.60	6.98	5.88	17.65
	Max.	0	5.88	16.67	20.22	17.88	31.58	55.56
$\beta_{yh}$	Min.	0	0	5.56	5.26	6.38	5.88	17.65
	Max.	0	0	5.88	12.09	13.85	17.65	41.18
α	Min.	0	0	5.88	7.32	8.22	11.76	23.53
	Max.	0	0	5.88	7.54	8.93	11.76	26.32

**Table 5:** Proportion of subclinically infected cows from the sensitivity analysis of the epidemiologically most significant parameters. The parameters and their values in the basic model and the sensitivity analysis are defined in Table 3. Percentile, PC; quantile, Q; standard deviation, SD.

Parameter	Min./Max. Value	5th PC	1st Q	Median	Mean	SD	3rd Q	95th PC
Basic model	/	0	0	0	4.00	5.42	5.88	15.79
σ	Min.	0	0	0	3.05	4.41	5.88	11.76
	Max.	0	0	0	3.46	4.8	5.88	11.76
ω	Min.	0	0	0	3.82	4.91	5.88	11.76
	Max.	0	0	0	4.17	5.47	5.88	15.79
$\beta_{i}$	Min.	0	0	0	3.98	5.46	5.88	15.79
	Max.	0	0	5.88	9.34	10.84	14.29	31.58
$\beta_{h}$	Min.	0	0	0	3.76	4.99	5.88	11.76
	Max.	0	0	0	3.76	4.99	5.88	11.76
P	Min.	0	0	0	2.32	4.33	5.56	11.76
	Max.	0	0	5.88	7.41	7.49	11.76	23.53
$\beta_{yi}$	Min.	0	0	0	3.40	4.75	5.88	11.76
	Max.	0	0	5.88	10.55	10.83	17.65	33.33
$\beta_{yh}$	Min.	0	0	0	3.19	4.50	5.88	11.76
	Max.	0	0	5.56	6.68	8.36	11.11	23.53
α	Min.	0	0	4.76	4.39	5.63	5.88	16.67
	Max.	0	0	0	4.09	5.50	5.88	16.67

**Table 6:** Proportion of clinically infected cows from the sensitivity analysis of the epidemiologically most significant parameters. The parameters and their values in the basic model and the sensitivity analysis are defined in Table 3. Percentile, PC; quantile, Q; standard deviation, SD.

Parameter	Min./Max. Value	5th PC	1st Q	Median	Mean	SD	3rd Q	95th PC
Basic model	/	0	0	0	0.47	1.63	0	5.56
σ	Min.	0	0	0	0.25	1.17	0	0
	Max.	0	0	0	0.33	1.34	0	5.56
ω	Min.	0	0	0	0	0	0	0
	Max.	0	0	0	0.24	1.19	0	0
$\beta_{i}$	Min.	0	0	0	0.45	1.61	0	5.56
	Max.	0	0	0	0.54	1.73	0	5.88
$\beta_{h}$	Min.	0	0	0	0.42	1.54	0	5.56
	Max.	0	0	0	0.42	1.54	0	5.56
P	Min.	0	0	0	0.27	1.23	0	0
	Max.	0	0	0	0.71	2.02	0	5.88
$\beta_{yi}$	Min.	0	0	0	0.40	1.49	0	5.56
	Max.	0	0	0	0.88	2.28	0	5.88
$\beta_{yh}$	Min.	0	0	0	0.39	1.49	0	5.56
	Max.	0	0	0	0.65	1.96	0	5.88
α	Min.	0	0	0	0.36	1.42	0	5.56
	Max.	0	0	0	0.57	1.80	0	5.88

## Data Availability

The data presented in this study and the developed model are available on request from the corresponding author.
